# Genetic and environmental influences on MRI scan quantity and quality

**DOI:** 10.1016/j.dcn.2019.100667

**Published:** 2019-05-27

**Authors:** Michelle Achterberg, Mara van der Meulen

**Affiliations:** a Leiden Consortium on Individual Development, Leiden University, the Netherlands; b Institute of Psychology, Leiden University, the Netherlands; c Leiden Institute for Brain and Cognition, Leiden University, the Netherlands

**Keywords:** Childhood, Functional MRI, Head motion, Heritability, Scanner related distress, Structural MRI

## Abstract

The current study provides an overview of quantity and quality of MRI data in a large developmental twin sample (N = 512, aged 7–9), and investigated to what extent scan quantity and quality were influenced by genetic and environmental factors. This was examined in a fixed scan protocol consisting of two functional MRI tasks, high resolution structural anatomy (3DT1) and connectivity (DTI) scans, and a resting state scan. Overall, scan quantity was high (88% of participants completed all runs), while scan quality decreased with increasing session length. Scanner related distress was negatively associated with scan quantity (i.e., completed runs), but not with scan quality (i.e., included runs). In line with previous studies, behavioral genetic analyses showed that genetics explained part of the variation in head motion, with heritability estimates of 29% for framewise displacement and 65% for absolute displacement. Additionally, our results revealed that subtle head motion (after exclusion of excessive head motion) showed lower heritability estimates (0–14%), indicating that findings of motion-corrected and quality-controlled MRI data may be less confounded by genetic factors. These findings provide insights in factors contributing to scan quality in children, an issue that is highly relevant for the field of developmental neuroscience.

## 1 Introduction

In the first decade of life, extensive changes occur in the structure and function of the brain (Gilmore et al., 2018). With the introduction of Magnetic Resonance Imaging (MRI), these changes in brain characteristics can be studied in vivo, and a growing body of literature has provided insight in the developing brain. Although MRI research is non-invasive, the scanner itself - in particular its noise level and narrow space- and the surrounding procedures are rather imposing and can induce anxiety in children (Durston et al., 2009; Tyc et al., 1995). Such scanner related distress makes it less likely for children to successfully finish an MRI scan, resulting in reduced scan quantity compared to older samples. Moreover, the quality of the scans heavily depends on the amount of (head) motion, which is specifically troublesome in developmental samples, as head movement during MRI is strongly correlated with age (Poldrack et al., 2002; Satterthwaite et al., 2013). Several prior developmental neuroimaging findings have been called into question after studies showed that these findings were largely influenced by age-related differences in head motion (Power et al., 2012; Savalia et al., 2017; Van Dijk et al., 2012), highlighting the need for an in-depth investigation of factors that can influence scan quality in children. In the current study we therefore provide an overview of MRI scan quantity and quality in a large developmental twin sample (N = 512, 256 twin pairs, aged 7–9), and investigated the genetic and environmental influences on MRI data quantity and quality.

Scan quality is not only influenced by head motion but can also be influenced by additional sources of noise such as scanner drift and respiratory signals (Kotsoni et al., 2006; Liu, 2017; Power, 2017). However, as excessive head motion is especially pronounced in developmental samples (Satterthwaite et al., 2013), the current study focused on head motion as measure of scan quality. In the last couple of years, the topic of MRI motion artifacts has received increasing attention, and several methods to correct for motion during MRI analyses have been developed (Fassbender et al., 2017; Power, 2017; Power et al., 2015). Much less research has focused on specific factors that contribute to MR scan quality in children. Recent studies have pointed towards genetics as a possible factor influencing scan quality, with findings suggesting that head motion in adults is a stable and heritable phenotype (Couvy-Duchesne et al., 2014; Van Dijk et al., 2012), with heritability estimates ranging from 37 to 51% in adults. Exploratory twin-analyses on pediatric MRI data also showed familial similarities in children (Engelhardt et al., 2017), although the small sample size hindered direct estimations of heritability. In the current study we provide direct estimates of heritability by conducting behavioral genetic analyses on a large childhood twin sample.

In addition to trait-like, genetic influences on scan quality, we also investigated the influence of environmentally affected factors, such as emotional state towards the MR scan and MR protocol length. Previous research has described several child-specific scanner environment adaptations that have been used in (clinical) radiology departments (Fassbender et al., 2017; Galvan et al., 2012; Raschle et al., 2012). One adaptation that has been shown to be particularly useful is the use of a mock scanner (Durston et al., 2009; Hallowell et al., 2008; Rosenberg et al., 1997), which replicates the MRI environment and can be used to familiarize young subjects with the procedure of an MRI scan. Children who underwent such an MRI simulation were less stressed (as indicated by lower heart rate) than children who were not trained with a simulator (Rosenberg et al., 1997). Moreover, studies showed a linear decrease in (self and parent reported) anxiety levels after MRI simulation (Durston et al., 2009; Rosenberg et al., 1997), indicating that an MRI simulation can make children feel more at ease with MRI research. This is important for the well-being of the participant, and a positive experience with the MRI scan can also increase retention of participants in longitudinal imaging studies, which is important for the validity of developmental MRI studies (Telzer et al., 2018). However, it is currently unknown whether a more positive emotional state towards the MRI scan is related to better outcomes in terms of scan quantity and quality. By using multi-informant estimations of emotional state, we directly tested the relation between scanner related distress and scan quantity and scan quality. We first examined how scanner related distress changed over time at three moments: before the MRI simulation, before the MRI scan, and after the MRI scan. We hypothesized that the emotional state would become more positive over time (Durston et al., 2009). Moreover, we hypothesized that there would be little influence of genetics on scanner related distress, as it is highly influenced by the environment (i.e., the MRI simulation). Next, we evaluated MRI scan quantity by investigating how scan quantity was related to emotional state, and to what extend scan quantity was influenced by genetics. Scan quantity was defined as the number of completed MRI runs within the protocol (ranging from 0 to 9). It should be noted that completing a run does not necessarily indicate that the MRI data is useable, and therefore scan quantity is essentially different from scan quality.

Similar to scan quantity, we investigated whether scan quality was related to emotional state, and to what extend scan quality was influenced by genetics. As an additional factor of interest, we examined scan quality across the duration of the MR session, as children tend to lose focus faster than adults, which may result in increased motion over time (Fassbender et al., 2017; Van Horn and Pelphrey, 2015). Scan quality was examined in two ways: 1) the percentage of included MRI runs within the session (defined as the number of scans with sufficient quality relative to the number of runs completed), and 2) the amount of absolute and framewise head displacement in mm in fMRI runs. The first estimate of scan quality provides an overall, relatively simple measure of quality over the whole MRI session. The second measure provides a more sophisticated, quantitative measure of scan quality, but could only be calculated for functional MRI runs (Power, 2017). By investigating both trait-like genetic influences as well as state-like environmental influences this study can provide insights in factors contributing to scan quantity and quality in developmental samples.

## 2 Methods

### 2.1 Participants

Participants in this study took part in the preregistered longitudinal twin study of the Leiden Consortium on Individual Development (L-CID; Euser et al. (2016)). The Dutch Central Committee on Human Research (CCMO) approved the study and its procedures (NL50277.058.14). Families with a same-sex twin born between 2006–2009, living within two hours travel time from Leiden, were recruited through municipal registries and received an invitation to participate via mail. Parents could show their interest in participation using a reply card. 512 children (256 families) between the ages 7 and 9 were included in the L-CID study (mean age: 7.94 ± .67; 49% boys). Written informed consent was obtained from both parents. All children were fluent in Dutch or English and had normal or corrected-to-normal vision. The majority of the sample was Caucasian (90%) and right-handed (87%). Since the sample represents a population sample, we did not exclude children with a psychiatric disorder. For information on psychiatric disorders, we asked parents whether the children received a medical diagnosis from a psychologist or medical expert. Eleven participants (2%) were diagnosed with an Axis-I disorder: nine with attention deficit (hyperactivity) disorder (ADD/ADHD); one with generalized anxiety disorder (GAD), and one with pervasive developmental disorder-not otherwise specified (PDD-NOS). Participants’ intelligence (IQ) was estimated with the subtests ‘Similarities’ and ‘Block Design’ of the Wechsler Intelligence Scale for Children, third edition (WISC-III; Wechsler (1991)). Estimated IQs were in the normal range (72.50–137.50, mean: 103.58 ± 11.76). Zygosity was determined by DNA analyses, which classified 55% of the twins as monozygotic.

### 2.2 Procedure

Participating twins visited the lab with their primary parent (defined as the parent that spends the most time with the children). Before the visit to the lab families received a step-by-step explanation of the MRI procedure, including a description of the magnetic field, the materials used during the MRI scan (earplugs, headphones, button box, alarm), and the movies that were available to watch. The step-by-step approach was specifically aimed at the young participants, and consisted of child appropriate texts and illustrative pictures. The lab visit took place at the Leiden University Medical Centre (LUMC) and consisted of four components: the MRI preparation session, the MRI scan session, parent-child interaction tasks, and a child behavioral tasks session. In the current study, data from the MRI preparation session and the MRI scan session were evaluated. During the practice session the whole family was further introduced to the aims of the study, and carefully instructed about safety around the MRI system and the influence of motion on the scans. Next, the children participated in a MRI simulation with the MRI researcher. In the MRI simulation, the exact same steps that were also explained in the step-by-step explanation were followed. A prototype of a Philips scanner (without a working magnet) was used to mimic the MRI environment. Children listened to MRI sounds via a laptop. They were shown the various materials (e.g. headphones, button box, coil with mirror attached) for the MRI procedure. Next, they were asked to practice lying very still on the scanner bed while wearing the headphones and button box. Finally, they practiced looking in the mirror on the coil, while they were slowly slid into the MRI bore. After the MRI simulation, the children were familiarized with the MRI tasks on a laptop. First-born and second-born children of each twin pair were randomly assigned to the MRI scan session or to the parent-child interaction tasks as their first activity. There were no differences in outcome measures (scanner related distress, scan quantity or scan quality) for children that were scanned directly after the MRI simulation or an hour later.

The MRI session lasted 60 min, including two fMRI tasks, high resolution T2 and T1 scans, diffusion tensor imaging (DTI) scans and a resting state (RS) fMRI scan. The first fMRI task was the Social Network Aggression Task (SNAT), as described in detail in Achterberg et al. (2018b). In short, participants viewed pictures of peers that gave positive, neutral or negative feedback to the participant’s personal profile. Next, participants could blast a loud noise towards the peer as an index of aggression. The SNAT consisted of 3 runs of approximately 5 min each. The second task was the Prosocial Cyberball Game (PCG), as described in detail in van der Meulen et al. (2018). In short, participants were instructed to participate in a virtual ball tossing game with three other players. During the game, two of the other players excluded the third player. The participant could choose to compensate for this exclusion by tossing the ball more often to the excluded participant (prosocial compensating behavior). The PCG consisted of 2 runs of approximately 5 min each. After the fMRI tasks participants watched a self-chosen child-friendly movie during the structural anatomical scan (3DT1) and the structural connectivity scans (DTI). The scan session ended with a RS fMRI scan, in which participants were instructed to lay still with their eyes closed and not to fall asleep (for details, see Achterberg et al. (2018a)). The order of the scans was the same for all participants and always started with the SNAT fMRI task, followed by the PCG fMRI task, the 3DT1, DTI and the RS fMRI.

### 2.3 Scanner related distress

**Fig. 1:**
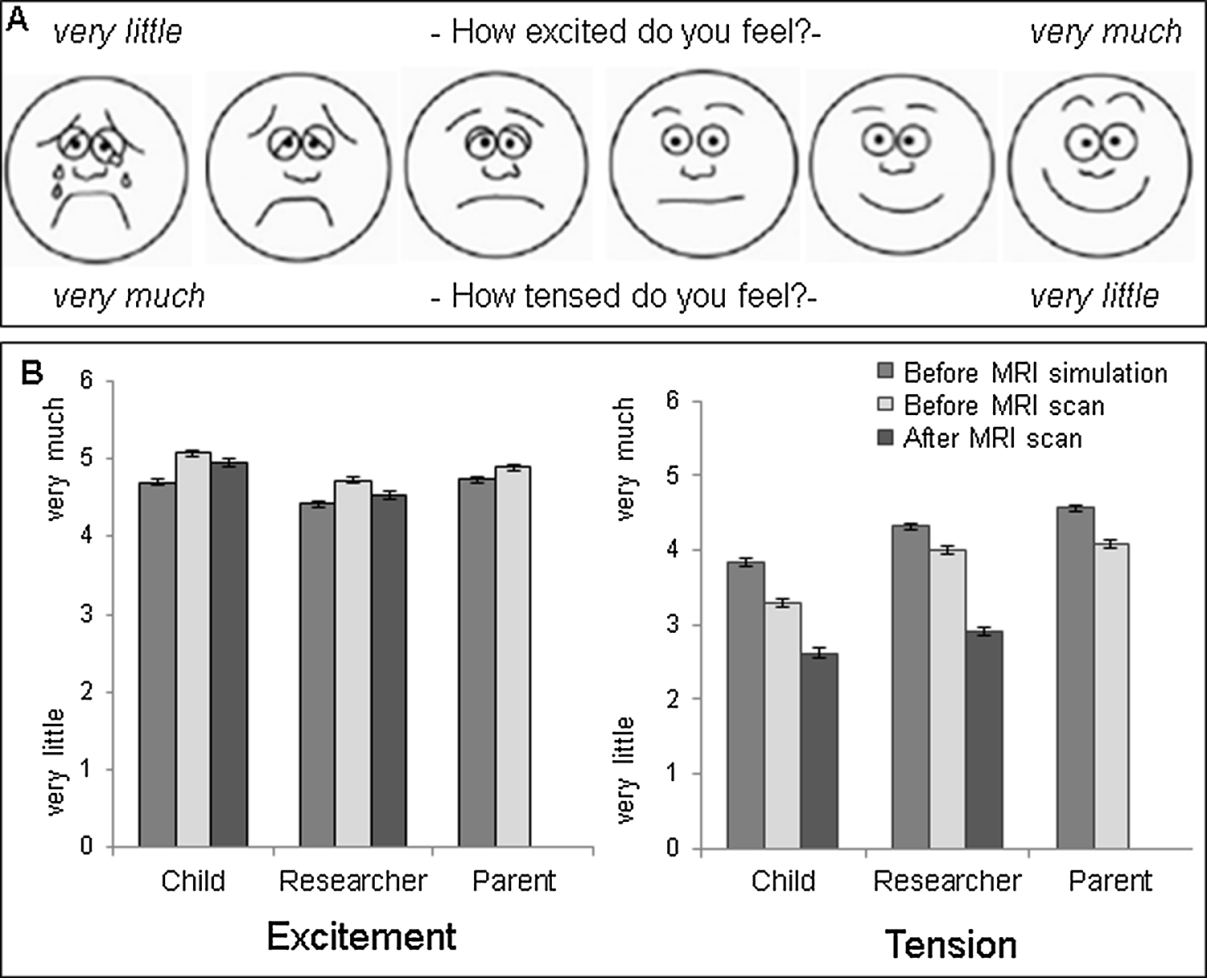
Emotional state towards the MRI scan. A) Visual analogue scales. B) Estimation of excitement and tension on three moments (before MRI simulation, before MRI scan, and after MRI scan) and by three raters (child, researcher, and parent).

To get an estimate of the children’s scanner related distress we asked the children to indicate how they felt about the scanner by using a visual analogue scale, based on Durston et al. (2009). Children’s feelings of stress and excitement were assessed at three different moments: before the MRI simulation, before the MRI scan, and after the MRI scan. Participants were asked to indicate how *tensed* and how *excited* they felt about the scan session, by pointing to the cartoon smiley that best represented their feelings (Fig. 1a). Since children tend to underreport their tension or anxiety (Durston et al., 2009), the child’s emotional state was consecutively also estimated by the researcher and the parent. It should be noted that both the child’s and the researcher’s estimates were written on the same form with the child reporting first, making them not independent. The parents estimated scanner related distress separately from the child and therefore these estimates were independent. Therefore, multi-informant ratings were based on child and parent reports. Parents, however, did not estimate the children’s emotional state after the MRI scan, as they were not present during the MRI scan (being involved in parent-child interaction tasks with the other twin sibling). Therefore, the scores after the MRI scan were based on child report only.

### 2.4 MRI data acquisition

MRI scans were acquired with a standard whole-head coil on a Philips Ingenia 3.0 T MRI system. To prevent head motion, foam inserts surrounded the children’s heads. The fMRI tasks and the movie were projected on a screen that was visible through a mirror on the head coil. Functional runs of the fMRI tasks (first task: SNAT (Achterberg et al., 2018b); second task: PCG (van der Meulen et al., 2018)) were acquired using a T2*-weighted echo-planar imaging (EPI). The first two (dummy) volumes were discarded to allow for equilibration of T1 saturation effects. The SNAT consisted of 3 runs in total with 148 volumes (5.43 min), 142 volumes (5.21 min), and 141 volumes (5.17 min) respectively. The PCG consisted of 2 runs in total. The number of volumes was dependent on the reaction time of the participant, with a maximum of 175 volumes. On average, 136 volumes (4.99 min) were acquired for each PCG run. Volumes covered the whole brain with a field of view (FOV) in mm = 220 (ap) x 220 (rl) x 111.65 (fh) mm; repetition time (TR) of 2.2 s; echo time (TE) =30 ms; flip angle (FA) = 80°; sequential acquisition, 37 slices; and voxel size = 2.75 × 2.75 x 2.75 mm. Subsequently, a high-resolution 3D T1scan was obtained as anatomical reference (FOV = 224 (ap) x 177 (rl) x 168 (fh); TR =9.72 ms; TE = 4.95 ms; FA = 8°; 140 slices; voxel size 0.875 × 0.875 x 0.875 mm). In addition, a high-resolution EPI scan was obtained for RS-fMRI registration purposes (TR =2.2 s; TE =30 ms, flip angle = 80°, FOV = 220.000 (rl) x 220.00 (ap) x 168.00 (fh), 84 slices). Next, two transverse Diffusion Weighted Imaging (DWI) scans were obtained with the following parameter settings (similar to Achterberg et al. (2016)): 30 diffusion-weighted volumes with different noncollinear diffusion directions with b-factor 1000 s/mm2 and 5 diffusion-unweighted volumes (b-factor 0 s/mm2); anterior -posterior phase encoding direction; parallel imaging SENSE factor = 3; flip angle = 90°; 75 slices of 2 mm; no slice gap; reconstruction matrix 128 × 128; FOV = 240 × 240 mm; TE = 69 ms; TR = 7315 ms. The second DWI set had identical parameter settings as used for the first set except that it was acquired with a reversed k-space readout direction (posterior-anterior phase encoding direction) enabling the removal of susceptibility artifacts during post processing (Andersson et al., 2003). Resting state data was acquired at the end of the imaging protocol (for details see Achterberg et al. (2018a)). A total of 142 T2 -weighted whole-brain echo planar images (EPIs) were acquired, including 2 dummy volumes preceding the scan to allow for equilibration of T1 saturation effects (TR =2.2 s; TE =30 ms; flip angle = 80°; FOV = 220.000 (rl) x 220.00 (ap) x 111.65 (fh); 37 slices).

### 2.5 MRI data quality control

Motion estimation of functional MRI (task-based and resting state) was carried out using Motion Correction FMRIB’s Linear Image Registration Tool (MCFLIRT Jenkinson et al. (2002), as implemented in the FMRIB Software Library (FSL) version 5.09 (Smith et al., 2004). Absolute displacement (AD) in x, y, and z direction was calculated for all runs, for all participants (Table 1), with the middle volume of the run as a reference. We additionally investigated micro-movement (i.e., motion between two volumes) using the motion outlier tool (*fsl_motion_outliers*). Mean framewise displacement (FD) was calculated for all runs, for all participants (Table 1). Reliability analyses showed consistency in head motion over fMRI runs: mean FD: α = .77; mean AD (mean x-y-z direction): α = .84. For further analyses we computed a mean score over all fMRI runs for framewise displacement (M = .77, SD = 1.29, range = .09–17.5) and absolute displacement (M = 2.55, SD = 3.77, range = .21–37.91). Framewise and absolute displacement were significantly positively correlated: *r=.*88, *p* < .001. For task-based fMRI runs, we defined runs with <3 mm (1 voxel) maximum motion in all directions as sufficient quality (Achterberg et al., 2018b; van der Meulen et al., 2018). For the RS fMRI data, volumes with framewise displacement of >0.3 mm (stringent threshold) or >0.5 (lenient threshold) were flagged as outliers (Power et al., 2012). RS fMRI data with <20% of the volumes flagged as outlier was classified as sufficient quality, see Table 1. Although inclusion criteria for task-based and RS fMRI were different, they resulted in comparable motion estimates for the different fMRI runs of included participants (Table 1).

**Table 1:** Framewise and absolute head displacement.

	N	meanFD (mm)	mean X, Y, Z (mm)	X (mm)	Y (mm)	Z (mm)
*all participants*						
SNAT run 1	488	.48 (1.12)	1.82 (4.37)	.59 (1.33)	2.27 (6.44)	2.59 (5.90)
SNAT run 2	483	.65 (1.28)	2.11 (4.38)	.71 (1.66)	2.65 (6.82)	3.00 (5.19)
SNAT run 3	481	.68 (1.05)	2.20 (3.64)	.78 (1.37)	2.52 (4.44)	3.30 (5.71)
PCG run 1	480	.69 (1.63)	2.37 (5.10)	.79 (1.83)	2.84 (7.53)	3.46 (6.43)
PCG run 2	478	.98 (3.23)	3.01 (5.50)	1.01 (1.89)	3.53 (8.78)	4.48 (7.26)
RS	442	1.07 (2.4)	3.82 (6.80)	1.16 (2.38)	4.47 (8.73)	5.83 (9.97)

*included participants*						
SNAT run 1^a^	385	.32 (.90)	.76 (.42)	.29 (.25)	.87 (.56)	1.11 (.66)
SNAT run 2^a^	345	.26 (.14)	.74 (.43)	.27 (.26)	.83 (.57)	1.10 (.68)
SNAT run 3^a^	320	.28 (.18)	.79 (.49)	.30 (.31)	.91 (.65)	1.17 (.76)
PCG run 1^a^	307	.24 (.14)	.72 (.44)	.25 (.25)	.83 (.58)	1.07 (.71)
PCG run 2^a^	266	.27 (.15)	.82 (.47)	.29 (.30)	.93 (.59)	1.24 (.79)
RS stringent^b^	151	.18 (.08)	.75 (1.26)	.23 (.24)	.79 (.63)	1.21 (3.35)
RS lenient^c^	230	.25 (.27)	1.04 (1.47)	.30 (.32)	1.15 (1.51)	1.68 (3.14)

a Based on <3 mm absolute displacement (X, Y and Z).

b Based on <20% frames with >0.3 mm framewise displacement.

c Based on <20% frames with >0.5 mm framewise displacement.

**Fig. 2:**
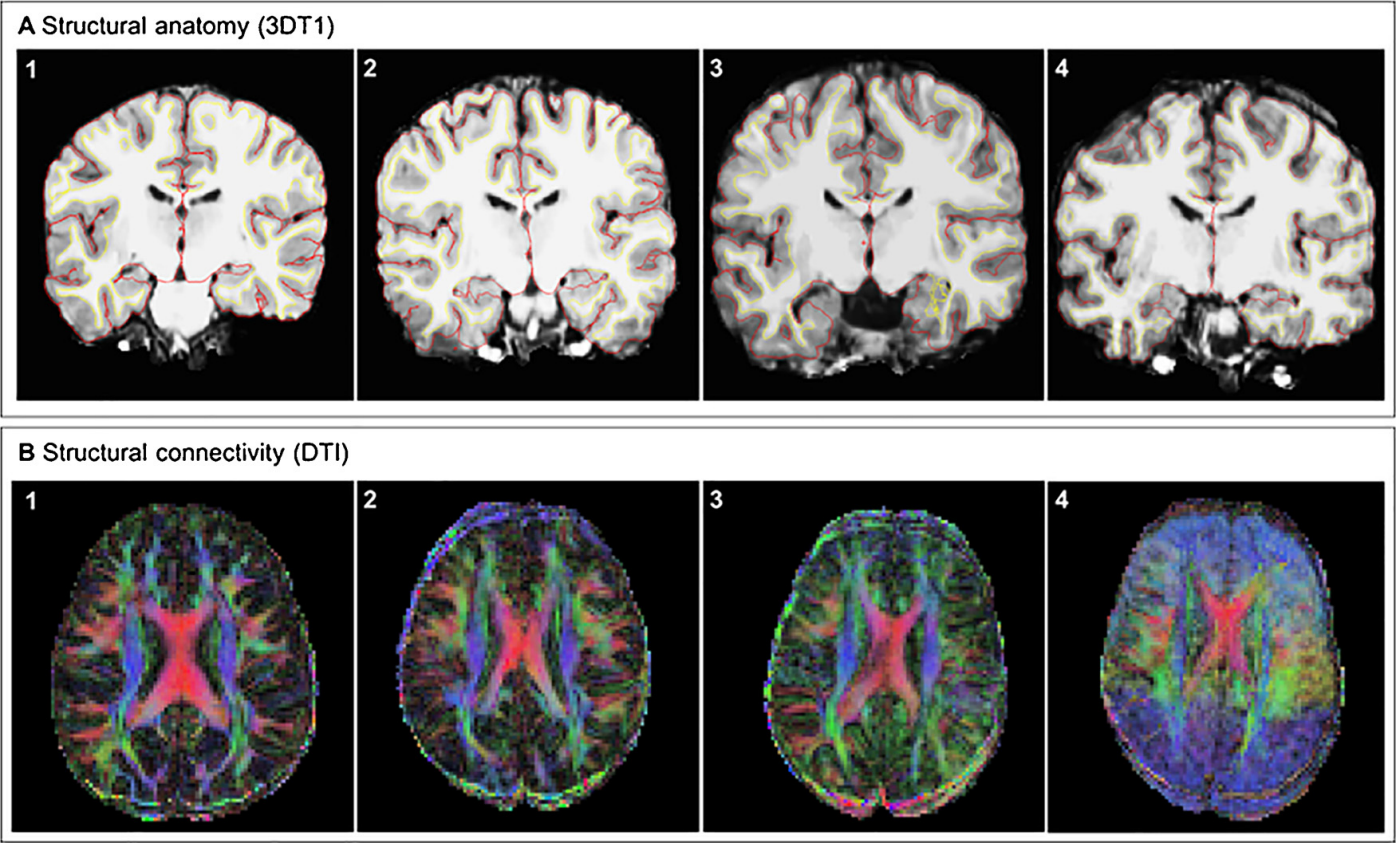
Examples of quality control classifications with scans rated as (1) Excellent, (2) Good, (3) Doubtful, and (4) Failed. A) Parcellated structural anatomy (T1 weighted) scan with pial surface (red line) and white matter/grey matter division (yellow line). B) Diffusion tensor fitted structural connectivity (DTI) scan with connections in right-left (red), anterior-posterior (green), and dorsal-ventral (blue) direction (For interpretation of the references to colour in this figure legend, the reader is referred to the web version of this article).

Structural T1 scans were pre-processed in FreeSurfer (v5.3.0). Anatomical labeling and tissue classification was performed on the basis of the T1- weighted MRI image using various tools of the FreeSurfer software (http://surfer.nmr.mgh.harvard.edu/). The pre-processing pipeline included non-brain tissue removal, cortical surface reconstruction, subcortical segmentation, and cortical parcellation (Dale et al., 1999; Fischl et al., 1999). After pre-processing, each scan was manually checked to assess quality by three trained raters. Scans were rated based on a set of specific criteria (e.g., affection by movement, missing brain areas in reconstruction, inclusion of dura or skull in reconstruction, see Klapwijk et al. (2019). 31% of the structural T1 scans were rated as ‘Excellent’, 43% of the scans were rated as ‘Good’, 16% of the scans were rated as ‘Doubtful’, and 10% of the scans were rated as ‘Failed’ (see Fig. 2a). Structural anatomical data rated as ‘Failed’ and ‘Doubtful’ were classified as insufficient quality, and data coded as ‘Excellent’ and ‘Good’ were classified as sufficient quality. We investigated whether scans with different ratings would show actual differences in estimated brain volume, by comparing the four different ratings on the “Total Gray Volume” variable from the Freesurfer output. We found a significant difference in gray matter volume between the different ratings (*F*(3, 463) = 5.07, *p* = .002), with post hoc analyses revealing a significant difference between scans rated as ‘Failed’ and scans rated as ‘Excellent’ to ‘Doubtful’ (all *p’s*<.02). Therefore, for analyses using more lenient quality control, we included data that were classified as ‘Excellent’, ‘Good’ and ‘Doubtful’.

Diffusion weighted images were preprocessed using several FSL analysis tools. Firstly, Top-up was used to estimate and correct susceptibility induced distortions (Andersson et al., 2003). Secondly, the Brain Extraction Tool (BET) was used to delete non-brain tissue from images of the entire head (Smith, 2002). Third, the Eddy tool was used to correct for eddy current-induced distortions and subject movement. Thereafter, a diffusion tensor model was fitted at each voxel by using the analysis-tool DTIFIT. Scans were rated by two independent researchers. 86% of the DTI data were rated as ‘Excellent’, 8% of the data were rated as ‘Good’, 4% of the data were rated as ‘Doubtful’, and 2% of the data were rated as ‘Failed’ (see Fig. 2b). DTI data rated as ‘Failed’ and ‘Doubtful’ were classified as insufficient quality, and all other data (‘Excellent’ and ‘Good’) were classified as sufficient quality. For analyses using more lenient quality control, we included data that were classified as ‘Excellent’, ‘Good’ and ‘Doubtful’.

### 2.6 Statistical analyses

Statistical analyses were performed in the Statistical Package for Social Sciences (SPSS version 24) and in R version 3.5.0 (R Core Team, 2015). Scanner related distress over time was examined with repeated measures ANOVAs in SPSS. Associations between emotional state, scan quantity, and scan quality were investigated using Pearson’s correlations (in SPSS). To estimate familial influences on our outcome measures we calculated Pearson within-twin correlations for monozygotic (MZ) and dizygotic (DZ) twin pairs. Similarities among twin pairs are divided into similarities due to shared genetic factors (A) and shared environmental factors (C), while dissimilarities are ascribed to unique environmental influences and measurement error (E). Behavioral genetic modeling with the OpenMX package (Neale et al., 2016) in R (R Core Team, 2015) was used to provide estimates of these A, C, and E components. The correlation of the shared environment (factor C) was set to 1 for both MZ and DZ twins, while the correlation of the genetic factor (A) was set to 1 for MZ twins and to 0.5 for DZ twins (see Fig. S1). The last factor, unique environmental influences and measurement error, was freely estimated. We calculated the ACE models for emotional state towards the MRI scan, scan quantity, and scan quality. High estimates of A indicate that genetic factors play an important role, whilst C estimates indicate influences of the shared environment. If the E estimate is the highest, variance in motion is mostly accounted for by unique environmental factors and measurement error. We first examined genetic influences on mean FD and mean AD for all scanned participants. Next, we examined the influence of genetics on moderate head motion, by excluding participants with excessive head motion (>1 mm mean FD, >3 mm mean AD). To investigate the effects of minimal head motion we only included participants with little head motion (<0.3 mean FD, < 1 mm mean AD).

## 3 Results

### 3.1 Scanner related distress

#### 3.1.1 Scanner related distress over time

To investigate scanner related distress preceding and following the MRI scan, we measured the emotional state towards the scanner using the visual analogue scales. Over time, children reported more excitement and less tension, see Fig. 1b. That is to say, children reported being significantly more excited before the MRI scan (*M=* 5.10, *SD=* .93), and after the MRI scan (*M=* 4.95, *SD=* 1.23), compared to before the MRI simulation (*M=* 4.72, *SD=* .94; *F* (491) = 23.25, *p* < .001, all Bonferroni corrected pair-wise comparisons *p* < .05). Furthermore, children reported significantly less tension before the MRI scan (*M=* 3.28, *SD=* 1.44), and after the MRI scan (*M=* 2.62, *SD=* 1.49), compared to before the MRI simulation (*M=* 3.84, *SD=* 1.28; *F* (491) = 124.65, *p* < .001, all Bonferroni corrected pair-wise comparisons *p* < .05). Ratings of tension by the researchers and parents showed a similar pattern (Fig. 1b) and were significantly correlated with ratings of children (*r*-range = .23–.80, see Table S1). Scanner related distress (before simulation and before the MRI scan, for excitement and tension) was more strongly correlated between children and researchers (*r*-range: .70–.80, Table S1), than between children and parents (*r*-range .23–.42, Table S1), but it should be noted that the child and researcher filled in the rating at the same form and therefore were not independent. The multi-informant scores (estimated emotional state averaged across child and parent) of tension and excitement were significantly negatively correlated: *r*=-.33, *p* < .001 before MRI simulation, and *r*=-.35, *p* < .001 before the MRI scan.

#### 3.1.2 Genetic influences on scanner related distress

To investigate genetic and environmental influences on scanner related distress, we calculated Pearson’s within-twin correlations for MZ and DZ twins and performed behavioral genetic analyses. Within-twin correlations for the multi-informant ratings of scanner related distress (tension and excitement; before MRI simulation and before MRI scan) were similar for MZ and DZ twins (*r_mz_* range = .24–.58; *r_dz_* range = .22–.48; all *p*’s<.05, see Table 2). Behavioral genetic analyses revealed that scanner related distress was mostly explained by environmental factors, both the shared environment (C-range = 23–47%) as well as the unique environment/measurement error (E-range = 45–77%), with little to no influence of genetics (A-range = 2–27%) (Table 2).

**Table 2:** Genetic modeling of emotional state towards the MRI scan.

Mood estimates		MZ	DZ		A²	C²	E²
**Excitement**							
Before MRI simulation	*r*	.50**	.48**	*ACE*	0.02	0.47	0.51
	*n*^a^	138	114	*95% CI*	0.00-0.38	0.15-0.57	0.40-0.62

Before MRI scan	*r*	.41**	.30**	*ACE*	0.09	0.27	0.63
	*n*^a^	135	113	*95% CI*	0.00-0.47	0.00-0.45	0.51-0.76

**Tension**							
Before MRI simulation	*r*	.58**	.39**	*ACE*	0.27	0.28	0.45
	*n*^a^	138	114	*95% CI*	0.00-0.62	0.00-0.55	0.36-0.57

Before MRI scan	*r*	.24**	.22*	*ACE*	0.00	0.23	0.77
	*n*^a^	134	113	*95% CI*	0.00-0.36	0.00-0.34	0.66-0.90

* p < .05.

** p < .001.

a Number of complete twin pairs.

### 3.2 MRI quantity

#### 3.2.1 Scan quantity

**Fig. 3:**
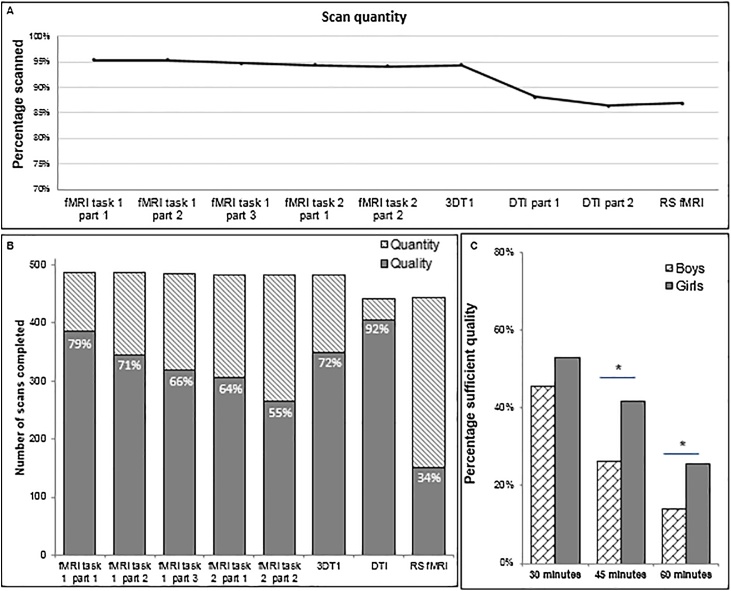
Scan quantity and quality. A) Scan quantity: the percentage of children that completed the MR run (100% = 512 participants). B) The number (and percentage) of scans with sufficient scan quality relatie to the quantity of the scans. C) Scan quality over time: the percentage of participants that were included on all scans in 30, 45 or 60 min, separately for boys and girls.

Of the 512 included participants, 24 children (4.7%) never started with the MRI scan due to MRI contra indications (n = 6); lack of parental consent (n = 4); technical error (n = 1), or substantial anxiety (n = 13), see Table S2. As can be seen in Table S2 and Fig. 3a, there was a drop in scan quantity (i.e. the number of scans completed) after the structural anatomy scan (from 94% to 88%). Scan quantity decreased because some children reported tiredness (n = 18) or due to time constraints (i.e. the reserved time was over; n = 12). For some children the DTI scans were skipped and only the RS-fMRI scan was acquired (n = 12), as the RS-fMRI run was shorter in duration (5 min compared to 2*5 min DTI). To investigate age and gender effects on scan quantity we compared participants who completed all scans (age *M=*7.96, *SD = 0.*67; 48% boys; n = 433), and participants who missed one or more scans (excluding participants who missed scans due to time constraints; age *M=*7.84, *SD = 0.*66; 59% boys, n = 39). However, we found no effects of age (*t*(470)=-1.08, *p* = .28) or gender (*χ*(1, N = 472) = 1.86, *p* = .12). We also found no association between age and the number of completed scans (*r* = .02, *p* = .63).

#### 3.2.2 Scan quantity in relation to scanner related distress

**Fig. 4:**
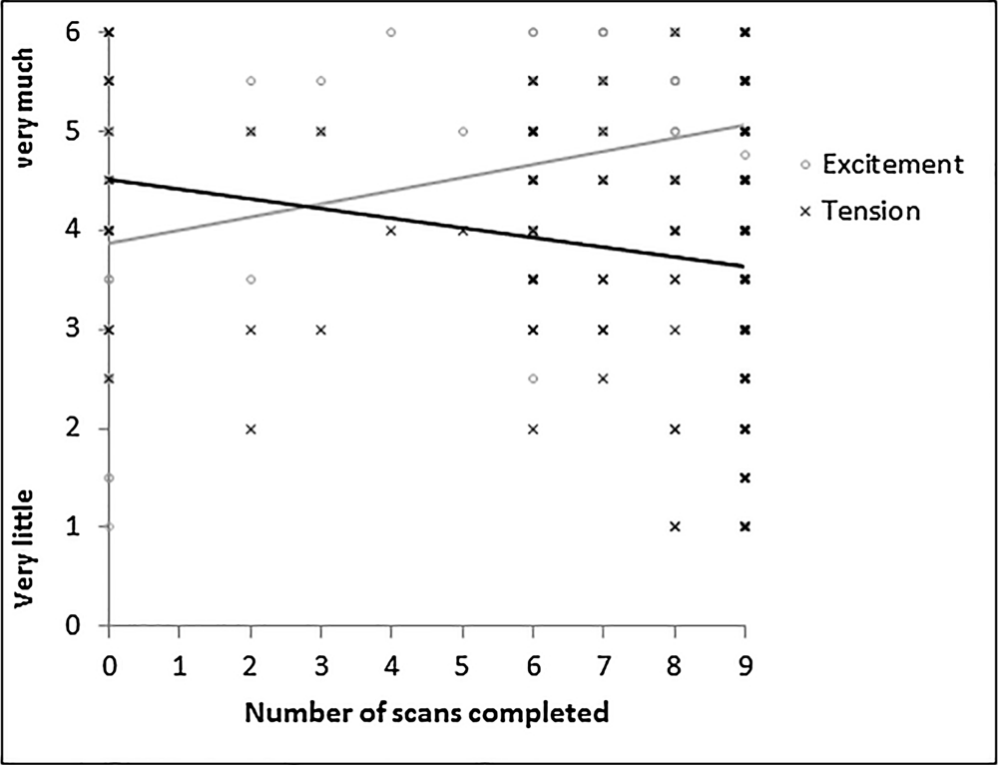
Number of scans completed plotted against excitement and tension towards the scan. Data visualized is from emotional state before MRI scan, emotional states before MRI simulation and after MRI scan showed similar patterns.

Pearson’s correlations on the number of completed scans (ranging from 0 to 9, *M=*8.29, *SD=*2.08) showed a positive association between excitement towards the scan and the number of scans completed (before MRI simulation: *r* = .21, *p* < .001; before MRI scan: *r* = .30, *p<*.001; after MRI scan: *r* = .25, *p* < .001), and a negative association between tension towards the scan and the number of scans completed (before MRI simulation: *r*=−.18, *p<*.001; before MRI scan: *r*=−.16, *p<*.001; after MRI scan: *r*=−.17, *p*<.001), see Fig. 4. All Pearson correlations were significant at Bonferroni corrected alpha level, adjusted for the number of distress estimates (six in total: excitement and tension before MRI simulation, before MRI scan, after MRI scan; α = 0.5/6, Bonferroni corrected α = .008).

#### 3.2.3 Genetic influences on scan quantity

To investigate genetic and environmental influences on scan quantity (number of scans completed), we calculated Pearson’s within-twin correlations for MZ and DZ twins and performed behavioral genetic analyses. Fisher r-to-z transformations showed that within-twin correlations for scan quantity were significantly stronger for MZ twins (*r_mz_* = .50, *p* < .001) than DZ twins (*r_dz_* = .14, *p* = .14), *Z* = 3.21, *p* < .001. Behavioral genetic analyses revealed substantial influences of genetics (A=45%, 95% CI [18–56%]) and unique environment/measurement error (E=55%, 95% CI [44–68%]), with no influence of the shared environment (C=0%, CI: 0–22%).

### 3.3 MRI quality

#### 3.3.1 Scan quality

An overview of the number (and percentage) of scans with sufficient quality relative to the quantity of the scans is provided in Fig. 3b. Of the 488 participants that started the MRI protocol, 385 participants (79%) had sufficient data in the first run. Sufficient MRI scan quality for task-based fMRI was defined as <3 mm (1 voxel) motion in all directions. The percentage sufficient data decreased over the first five task-based fMRI runs: 71% in the second run; 66% in the third run; 64% in the fourth run; and 55% in the fifth run. For the 3DT1 structural anatomy scans, 72% of the scans were classified as sufficient quality using a stringent threshold, and 88% was included using a lenient threshold (including scans coded as ‘Doubtful’). The percentage of DTI scans classified as sufficient quality was 92% using a stringent threshold and 96% using a lenient threshold (including ‘Doubtful’). The RS-fMRI data, which was the final run of the MRI session, showed the lowest scan quality, with 34% of the acquired data being of sufficient quality with a cut-off of <0.3 mm FD in > 20% of the volumes (Fig. 3b). Using a more lenient cut-off of <0.5 mm FD in > 20% of the volumes, 52% of the acquired data would have been included. Inclusion based on <3 mm absolute displacement (similar to the threshold used for task-based fMRI data) resulted in 51% of sufficient RS fMRI data. Across all scans, we found a small positive association between percentage of the acquired data being of sufficient quality (using stringent thresholds) and age (*r* = .10, *p* = .03).

#### 3.3.2 Scan quality over time

There was an increase in head motion over time, both framewise as well as absolute (x, y, and z-direction) displacement (Table 1). After excluding participants with insufficient data, head motion within the different task based and resting state fMRI runs was comparable (Table 1). To provide an overview of scan quality with respect to time, we calculated the percentage participants with sufficient quality data after 30, 45 and 60 min (for participants that completed the full scan protocol, n = 433, 48% boys), see Fig. 3c. The first 30 min consisted of four task-based fMRI runs; the 45 min included all task-based fMRI runs and the 3DT1. The 60-minute protocol was the full L-CID scan protocol. 214 participants (49%) had sufficient quality on all scans in the first 30 min, with no significant gender differences (*p* = .149), see Fig. 3c. 160 participants (33%) had sufficient quality on all scans in the first 45 min, with a larger proportion of girls being included than boys being included (*χ^2^*(1, N = 433) = 11.70, *p=*.001), see Fig. 3c. 87 participants (20%) had sufficient quality on all eight scans of the full 60-min protocol, with a larger proportion of girls being included than boys being included (*χ^2^*(1, N = 433) = 8.85, *p* = .002), see Fig. 3c. There were no age differences in scan quality over time.

#### 3.3.3 Scan quality in relation to scanner related distress

Pearson’s correlations on the number of included scans (range = 0–8, *M=*5.58, *SD=*2.47,) showed no association with excitement or tension (neither before the MRI simulation nor before the MRI scan, all *p’s*>.05). Children’s own estimate of excitement after the MRI scan was significantly correlated to scan quality (*r* = .13, *p* = .003), whereas tension after the MRI scan was not related to scan quality (*r* = .03, *p* = .52). Pearson’s correlations of the quantitative measures of scan quality (i.e. head motion based on the fMRI runs) showed a positive correlation between excitement before the MRI scan and mean FD (*r=.*12, *p=.*01), a positive association between absolute displacement and excitement before the MRI simulation (*r=.*10, *p=.*03) and before the MRI scan (*r=.*09, *p=.*04); and a negative association between absolute displacement and tension before the MRI simulation (*r=*-.09, *p=.*04). However, these correlations did not survive Bonferroni correction (Bonferroni corrected α = .008).

#### 3.3.4 Genetic influences on scan quality

Within-twin correlations for general scan quality (percentage of scans included) were significantly stronger for MZ twins (*r_mz_* = .47, *p* < .001) than DZ twins (*r_dz_* = .19, *p* = .05), *Z* = 2.40, *p* = .016. Behavioral genetic analyses revealed substantial influence of genetic factors (A=46%, 95% CI [33–58%]) and unique environment/measurement error (E=54%, 95% CI [42–67%]), with no influence of shared environment (C=0%, 95% CI [0–26%]).

Next, we investigated genetic influences on head motion, quantified by the mean framewise and mean absolute displacement over all fMRI runs. Within-twin correlations for framewise displacement were significantly stronger for MZ twins than DZ twins (*r_mz_* =.51, *p* < .001; *r_dz_*  = .19, *p* = .05, *Z* = 2.81, *p* = .002), see Table 3. Similar correlations were found for absolute displacement, with a significantly stronger association between MZ twins (*r_mz_* =.70, *p* < .001) than between DZ twins (*r_dz_* =.17, *p* = .09, *Z* = 5.27, *p* < .001), indicating substantial genetic influences. More detailed behavioral genetic analyses showed that framewise displacement was significantly influenced by genetics, with a heritability estimate of 29% (95% CI: [23–46%], Table 3). Absolute displacement also showed influence of genetics, with a heritability estimate of 65% (95% CI: [54–73%]), see Table 3.

**Table 3:** Genetic modeling of framewise and absolute head displacement for all participants scanned (prior to motion exclusion, including excessive head motion); for participants with moderate head motion (excluding excessive head motion); and for participants with minimal head motion (after stringent quality control).

Max motion		MZ	DZ		A²	C²	E²
**Excessive head motion**							
Framewise Displacement	*r*	.51**	.19	*ACE*	0,29	0,05	0,66
	*n*^a^	129	108	*95% CI*	0.00-0.46	0.00-0.39	0.54-0.80
Absolute Displacement	*r*	.70**	.17	*ACE*	0,65	0,00	0,35
	*n*^a^	129	108	*95% CI*	0.54-0.73	0.00-0.12	0.27-0.46

**Moderate head motion**^b^							
Framewise Displacement	*r*	.29**	.28*	*ACE*	0,12	0,22	0,66
	*n*^a^	96	72	*95% CI*	0.00-0.52	0.00-0.45	0.48-0.84
Absolute Displacement	*r*	.29**	.23	*ACE*	0,14	0,15	0,71
	*n*^a^	92	67	*95% CI*	0.00-0.46	0.00-0.39	0.54-0.90

**Minimal head motion**^c^							
Framewise Displacement	*r*	.26	.49*	*ACE*	0,00	0,33	0,67
	*n*^a^	28	18	*95% CI*	0.00-0.47	0.00-0.54	0.46-0.94
Absolute Displacement	*r*	.32	.28	*ACE*	0,06	0,29	0,65
	*n*^a^	24	20	*95% CI*	0.00-0.61	0.00-0.55	0.39-0.95

* p < .05.

** p < .001.

a Number of complete twin pairs.

b Mean FD < 1 mm; mean AD < 3 mm.

c Mean FD < 0.3 mm; mean AD < 1 mm.

**Fig. 5:**
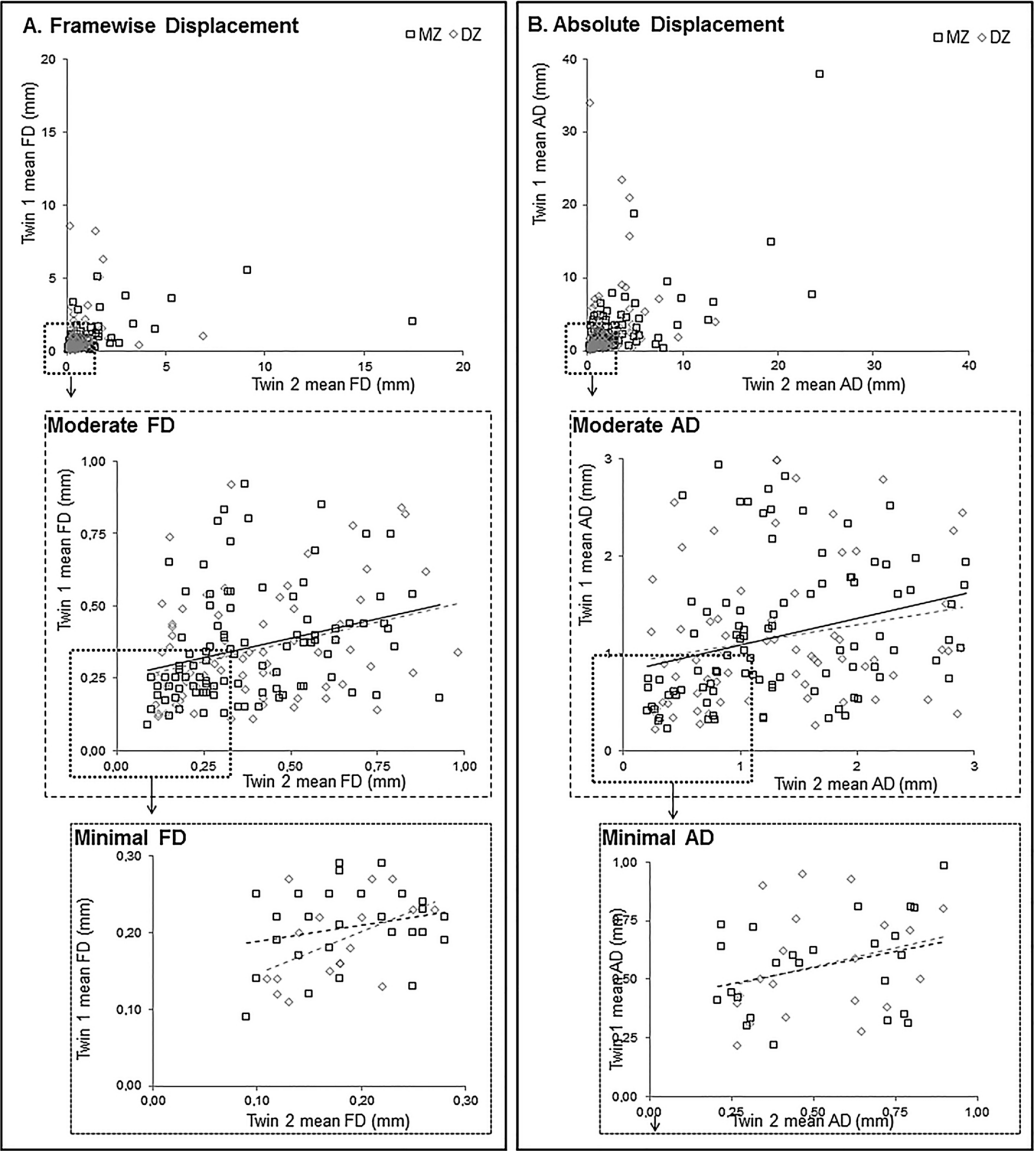
Visual representation of within-twin correlations of maximum head movement (in mm), split out by zygosity. A) Framewise head displacement in mm. The dashed frameworks are zoomed in on moderate (<1 mm mean FD) and minimal (<0.3 mm mean FD) head motion. B) Absolute head displacement in mm. The dashed frameworks are zoomed in on moderate (<3 mm mean AD) and minimal (<1 mm mean AD) head motion. Solid lines represent significant correlations (*p* < .001).

As is often the case with childhood samples, some participants displayed excessive head motion: up to 18 mm mean framewise displacement (Fig. 5a) and 38 mm mean absolute displacement (Fig. 5b). To prevent the genetic analyses from being biased by these extremes, we also investigated heritability of “moderate” head motion (Fig. 5). For these analyses, we only included participants with mean framewise displacement <1 and <3 mm absolute displacement. Within-twin correlations for moderate framewise displacement were similar for MZ twins (*r_mz_* = .29, *p* = .005) and DZ twins (*r_dz_*  = .28, *p* = .02, see Table 3). Similarly, within-twin correlation for moderate absolute displacement were similar for MZ twins (*r_mz_* = .29, *p* = .005) and DZ twins (*r_dz_* =.23, p = .06). Behavioral genetic analyses revealed low heritability estimates for moderate head motion (compared to overall head motion), and in addition showed influence of shared environment. That is to say, influence of genetics on moderate framewise displacement was estimated as 12% (95% CI: [0-51%]) and 22% of the variation was explained by shared environment (95% CI: [0–45%]). Influence of genetics on moderate absolute displacement was 14% (95% CI: [0–46]), and 15% of the variation was explained by shared environment (95% CI: [0–39%], Table 3).

As previous studies showed the tremendous effect of motion on fMRI signals in pediatric samples (Poldrack et al., 2002; Satterthwaite et al., 2013), and recent studies advise more stringent quality control (Power et al., 2014, 2015) we performed additional analyses on “minimal” head motion (Fig. 5). For these analyses, we only included participants with mean framewise displacement <0.3 mm and <1 mm absolute displacement. Within-twin correlations for minimal framewise displacement did not differ for MZ twins (*r_mz_* = 0.26, *p* = .183) and DZ twins (*r_dz_* =.49, *p* = .04; *Z*=-0.83, *p* = .406, see Table 3). Similarly, within-twin correlation for minimal absolute displacement were similar for MZ twins (*r_mz_* = .32, *p* = .123) and DZ twins (*r_dz_*  = .28, *p* = .225; *Z* = 0.14, *p* = .888). Behavioral genetic analyses revealed even lower heritability estimates for minimal head motion (compared to overall and moderate head motion, see Table 3). There was no influence of genetics on minimal framewise displacement (A = 0.00, 95% CI: [0–47%]) and 33% of the variation was explained by shared environment (95% CI: [0–54%]). Influence of genetics on minimal absolute displacement was 6% (95% CI: [0–61]), and 29% of the variation was explained by shared environment (95% CI: [0–55%]). Note that the sample size for analyses on minimal head motion was considerably smaller (n = 44 twin pairs, 55% MZ) than for analyses on moderate (n = 159 twin pairs, 59% MZ) and excessive (n = 237 twin pairs, 54%MZ) head motion. Fig. 5 provides a visual representation of the within-twin correlation of extreme, moderate and minimal head displacement, split out by zygosity.

## 4 Discussion

To address questions on quality of MRI scans in developmental samples we provided an overview of scan quantity and scan quality in a large developmental twin sample (N = 512 7-9-year-olds). Overall, scan quantity was high and 88% of the children completed all runs. We report a drop in the number of runs completed after approximately 45 min of scan time, which is comparable with prior findings in this age range (Engelhardt et al., 2017). Scan quality decreased with increasing scan time, consistent with previous studies that reported an increase in head motion over time (Centeno et al., 2016; Engelhardt et al., 2017; Fassbender et al., 2017).

### 4.1 Genetic influences on scan quantity and quality

As a complement to the growing literature on familial similarities in head motion (Couvy-Duchesne et al., 2014; Engelhardt et al., 2017), we also investigated genetic and environmental influences on scan quantity and scan quality. Behavioral genetic modeling showed substantial to strong heritability estimates (45–46%) for both scan quantity (number of runs completed) and scan quality (percentage of scans included). Whether or not a scan was included was based on often used, but arbitrary cut-off of head motion (task fMRI: <3 mm absolute head displacement; structural scans: manual ratings; RS-fMRI: <20% volumes with >0.3 mm framewise displacement). Therefore, we additionally estimated genetic influences of MRI scan quality on a more sophisticated and continuous measure of scan quality, i.e., the quantitative measures of head motion for all fMRI runs (framewise and absolute displacement in mm) including all scanned participants. Head motion over fMRI runs was stable (α = .77–84) and within-twin correlations were higher in MZ than DZ twins. Similar findings were previously reported by Engelhardt et al. (2017), showing familial similarity of pediatric framewise head displacement in RS-fMRI. To provide direct estimates of the percentage of variation explained by genetics and (shared and unique) environment, we used behavioral genetic analyses. These analyses revealed that head motion in fMRI runs was substantially influenced by genetics, with heritability estimates ranging from 29 to 65%, consistent with heritability estimates in adults (Couvy-Duchesne et al., 2014). Thus, both the overall measure of scan quality (percentage of scans included), as well as the more sophisticated measure of scan quality in fMRI runs (framewise- and absolute displacement) showed substantial influence of genetics. Together, these findings show evidence for genetic contributions to head motion, highlighting the need for careful control of motion related artifacts (Caballero-Gaudes and Reynolds, 2017; Power, 2017), specifically for studies in domains where genetic effects might play a strong role, such as in the case of psychiatric disorders that have a genetic basis (Hyman, 2000).

Reassuringly, heritability estimates for subtle head motion (after exclusion based on excessive head motion) were considerably smaller, ranging from 0 to 14%. This is contrary to previous findings of Engelhardt et al. (2017) who reported similar within-twin correlations on framewise head displacement before and after scrubbing (i.e., exclusion of frames with excessive motion). Differences might be due to the smaller sample size (N_mz_ = 12 and N_dz_ = 22) and the differences in exclusion based on head motion, since Engelhardt et al. (2017) excluded volumes with excessive head motion, whereas we excluded complete runs of participants with excessive head motion. Thus, in line with previous studies (Couvy-Duchesne et al., 2014; Engelhardt et al., 2017; Van Dijk et al., 2012), we report that excessive head motion is heritable and systematic, but additionally show that, after careful motion correction and exclusion based on excessive head motion, subtle head motion shows little influence of genetics. Possibly, subtle head movement is more strongly dependent on participant instruction and scanner adjustments. Indeed, behavioral genetic analyses on quality controlled head motion not only revealed small heritability estimates (0–14%, compared to 29–65% in overall head motion), but also showed that a similar, or even larger, proportion of the variance was explained by shared environmental influences (15–33%).

### 4.2 Environmental influences on scan quantity and quality

An additional goal of this study was to examine how emotional state towards the scanner was related to scan quality and quantity. Consistent with findings for quality controlled head movement, reports of emotional states showed little to no influence of genetics, but a moderate to strong relation with shared environmental influences. These findings suggest that emotional states can be significantly influenced by preparation of the scanner experiences. It was interesting to note that children’s tension was on average rated higher by researchers and parents than by children themselves, which is in line with previous studies suggesting that children may underreport their anxiety (Durston et al., 2009; Tyc et al., 1995). Multi-informant estimates of children’s emotional state towards the MRI scan were significantly associated with MRI quantity, as we found that children with higher estimated excitement and lower estimated tension completed more runs during the MRI scan. However, the association between children’s emotional state towards the MRI scan and scan quality was less clear, as the correlations did not survive Bonferroni correction. These findings suggest that by decreasing scanner related distress researchers can increase scan quantity, but more detailed future studies are necessary to reveal whether this would also lead to an increase in scan quality.

One aspect that did show influence on scan quality was the length of the MRI scan session. Results showed that a protocol of >30 min resulted in less than 50% sufficient quality on all scans in this age range of 7-9-year-olds. This is in line with other research that also recommends a scanning time of 30–40 min for young children (Raschle et al., 2012), whereas a longer scanning protocol of 60 min is only recommended for an older population ((Fassbender et al., 2017). If more scanning time is required to collect all data, a way to ensure scan quality would be to conduct two separate MRI sessions divided over different days (Fassbender et al., 2017). Moreover, as the field of (developmental) neuroimaging is rapidly evolving, the technology of MRI is progressing. New methods such as simultaneous multi-slice imaging (SMS or ‘Multiband’, Demetriou et al. (2018); Feinberg and Yacoub (2012)) and real-time monitoring of head motion (Framewise Real-time Integrated MRI Motion Monitoring (FIRMM; Dosenbach et al. (2017)) have the potential to drastically shorten acquisition time without compromising on the number of scans. The effects on these methods on MRI scan quality should be examined in more detail in future studies. For example, a pioneering study of Greene et al. (2018) reported that real time feedback about motion (using FIRMM) reduced head displacement in 5–10 year old children, but not in children older than 10.

### 4.3 Limitations

The study had several limitations, which should be addressed in future research. First, the current study examined one general aspect of scan quality (head motion), nevertheless, several other factors can influence scan quality, amongst others: thermal noise, respiratory signals, and scanner drifts (Kotsoni et al., 2006; Liu, 2017; Power, 2017). Future studies should also investigate the effects of these other factors, for example by investigating fMRI signal variability in regions of interest. Second, due to ethical considerations all participating children in the current study received the MRI simulation, therefore we were unable to directly test the effects of the MRI simulation and can only conclude that scanner related distress changed over time. Third, we report that children displayed the most head motion in the RS fMRI run, but this might be influenced by different definitions of sufficient quality, as the threshold for RS fMRI data was more conservative than the criteria for task-based fMRI. Nevertheless, Engelhardt et al. (2017) also report that their sample of 7-8-year-olds showed the most movement during rest and the least movement during an inhibition task and they suggested that the inhibition task was more engaging and therefore might have resulted in less head motion than the RS fMRI run. The sequence of MRI runs in our MR session was fixed, hindering direct comparison of task engagement, as the differences in head motion between task-based and RS fMRI might reflect a time effect. Studies in adults have indeed reported less head motion under engaging task conditions than during rest, irrespective of acquisition order (Huijbers et al., 2017) and future studies should investigate the effects of task demands versus time on scan quality in children. Relatedly, we instructed participants to lie still with eyes closed for the RS-fMRI. During the piloting phase of the scan protocol we experienced that the eyes closed condition was more comfortable for children than eyes open. Although recent studies have shown similar RS networks across different RS conditions (Yan, C. G., et al. (2009); Zou, Q., et al. (2015), differences in connectivity strength (Van Dijk et al., 2012; Yan, C. G., et al. (2009) and test-retest reliability have also been reported (Patriat, R., et al. (2013); Zou, Q., et al. (2015). Moreover, despite the specific instructions to participants to not to fall asleep, sleep was not directly monitored, which is a limitation of our RS design. Last, the behavioral genetic analyses had smaller sample sizes for **moderate** (N_mz_ = 92, N_dz_ = 67) and minimal head motion (N_mz_ = 24, N_dz_ = 20) than the analyses on the full sample (N_mz_ = 129, N_dz_ = 108). As the statistical power of genetic studies is influenced by the sample size (Verhulst, 2017), differences in results could be influenced by differences in sample sizes.

### 4.4 Conclusion

We report that participants’ scanner related distress was associated to scan quantity, but not to scan quality. Overall, scan quantity was high, as 88% of the children that started the protocol also completed it. The percentage of sufficient scans was considerably higher (49%) in the first 30 min of the protocol than in the full 60-minute protocol (20%), indicating that shorter scan protocols have less attrition. Consistent with previous studies (Couvy-Duchesne et al., 2014; Engelhardt et al., 2017), the behavioral genetic analyses revealed heritability effects on head motion, with heritability estimates ranging from 29 to 65%. Importantly, however, our results also show that after exclusion based on excessive head motion, heritability estimates declined to 0–14%, indicating that MRI findings of motion corrected and quality-controlled data are not substantially confounded by genetic factors. Moreover, shared environmental influences played a larger role (15–33%) in the variation in quality controlled head motion, suggesting that head motion can be influenced by participant instruction and scanner adjustments. These results provide insight in the genetic and environmental influences on scan quantity and quality and can inform future studies on developmental neuroimaging.
