# Supplementary material for: Genetic and environmental influences on MRI scan quantity and quality
Source: Dev Cogn Neurosci. 2019 May 27;38:100667. doi: 10.1016/j.dcn.2019.100667 (PMC6969338; doi:10.1016/j.dcn.2019.100667)
Supplement: Supplementary file 1 [file mmc1.docx]

**Genetic and environmental influences on MRI scan quantity and quality**

**Short title: Genetic influences on scan quality**

Michelle Achterberg, MSc ^1,2,3^ & Mara van der Meulen, MSc ^1,2,3^

**SUPPLEMENTARY FILES**

**Affiliations:**

^1^ Leiden Consortium on Individual Development, Leiden University, the Netherlands

^2^ Institute of Psychology, Leiden University, the Netherlands

^3^ Leiden Institute for Brain and Cognition, Leiden University, the Netherlands

**Corresponding author**: Michelle Achterberg, Faculty of Social and Behavioral Sciences, Leiden University, Wassenaarseweg 52, 2333AK Leiden, The Netherlands. Tel: +31 71 527 6861, E-mail: m.achterberg@fsw.leidenuniv.nl

**
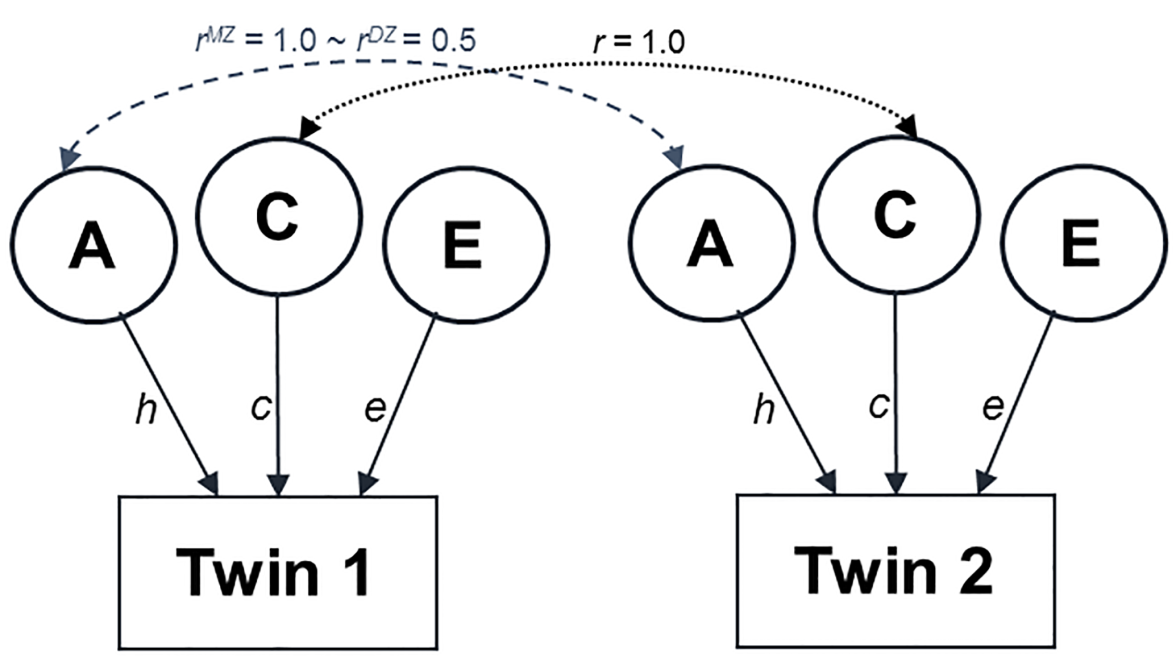
**

**Figure S1.** ACE model. Similarities among twin pairs are divided into similarities due to shared genetic factors (A) and shared environmental factors (C), while dissimilarities are ascribed to unique environmental influences and measurement error (E). The correlation of the shared environment (factor C) was set to 1 for both MZ and DZ twins, while the correlation of the genetic factor (A) was set to 1 for MZ twins and to 0.5 for DZ twins (see Figure S1). The last factor, unique environmental influences and measurement error, was freely estimated.

**Table S1.** Correlation matrix of emotional state towards the MR estimated by children, researchers, and parents.

|  |  | Children | |
| --- | --- | --- | --- |
|  |  | Excitement | Tension |
| Before MRI simulation | Researchers | .75** | .72** |
|  | Parents | .38** | .23** |
| Before MRI scan | Researchers | .80** | .70** |
|  | Parents | .42** | .35** |
| After MRI scan⁰ | Researchers | .73** | .74** |
| *⁰ Parents did not estimate the emotional state after the real scan* | | | |
| *** p<.001* |  |  |  |

**Table S2.** Scan quantity: percentage included participants and drop out reasons per MR run.

|  | fMRI task 1 part 1 | fMRI task 1 part 2 | fMRI task 1 part 3 | fMRI task 2 part 1 | fMRI task 2 part 2 | 3DT1 structural | DTI part 1 | DTI part 2 | RS fMRI | All scans completed |
| --- | --- | --- | --- | --- | --- | --- | --- | --- | --- | --- |
| Scan completed | 488 | 488 | 485 | 483 | 482 | 483 | 451 | 442 | 445 | 433 |
| *% completed* | *95%* | *95%* | *95%* | *94%* | *94%* | *94%* | *88%* | *86%* | *87%* | *85%* |
| Scan missing | 24 | 24 | 27 | 29 | 30 | 29 | 61 | 70 | 67 | 79 |
| *contra indication* | *6* | *6* | *6* | *6* | *6* | *6* | *6* | *6* | *6* | *6* |
| *no parental consent* | *4* | *4* | *4* | *4* | *4* | *4* | *4* | *4* | *4* | *4* |
| *technical error* | *1* | *1* | *1* | *1* | *1* | *1* | *1* | *1* | *1* | *1* |
| *Anxiety* | *13* | *13* | *14* | *14* | *15* | *15* | *17* | *17* | *19* | *19* |
| *child tired* | *-* | *-* | *2* | *2* | *2* | *2* | *20* | *29* | *23* | *33* |
| *time constraints* | *-* | *-* | *-* | *2* | *2* | *1* | *13* | *13* | *14* | *16* |
